# Laser-assisted p-PANI/n-CdS/p-Si dual-heterojunction architecture for UV/Vis/NIR wavelength detection

**DOI:** 10.1039/d6na00662k

**Published:** 2026-09-28

**Authors:** Shahad Raji, Ethar Yahya Salih, Asaad A. H. AlZubaidi, Mohammed Haider Sami

**Affiliations:** a Al-Karkh University of Science Baghdad 10081 Iraq; b College of Energy and Environmental Sciences, Al-Karkh University of Science Baghdad 10081 Iraq ethar988@gmail.com ethar@kus.edu.iq; c Technical College of Engineering, Al-Bayan University Baghdad Iraq

## Abstract

In this article, a novel p-PANI/n-CdS/p-Si dual-heterostructured photodetector is constructed using combined pulsed-laser-deposited CdS and localized laser-assisted coated PANI, enabling binary independently photo-active interfaces in a single heterojunction design. The microstructural and optical examination demonstrated successful CdS and PANI formation with optical bandgaps of 2.37 eV and 1.67 eV, respectively. The intended dual-junction design revealed pronounced rectification profiles, with ratios of 9.1 and 6.7 for p-PANI/n-CdS and n-CdS/p-Si, respectively. The former exhibited an enhanced photo-detection mechanism at 460 nm with responsivity and detectivity of 45.82 mA W^−1^ and 8.5 × 10^10^ Jones, respectively, while the latter demonstrated values of 417.75 mA W^−1^ and 2.58 × 10^11^ Jones, respectively, at 530 nm, along with remarkable performance in the NIR region (808 nm) owing to the p-Si wafer contribution. Both considered heterostructures exhibited excellent linear power dependency, with *R*^2^ ≈ 1. The dual-heterostructure demonstrated a rapid switching profile, with rise/fall times of 79/88 ms and 82/87 ms, respectively; the operational durability of the intended device revealed outstanding performance, retaining 93.7% and 87% for p-PANI/n-CdS and n-CdS/p-Si, respectively, over four weeks. The synergistic operation of the dual-heterostructure enables effective carrier separation and wavelength selection in the UV, Vis, and NIR regions, allowing an effective workflow for emerging high-performance multi-wavelength photo-detection based on hybrid organic/inorganic semiconductor design.

## Abstract 2

A laser-assisted p-PANI/n-CdS/p-Si dual-heterojunction architecture integrates two independently addressable photoactive interfaces, enabling complementary wavelength-selective photodetection across the UV-visible-NIR spectrum.

## Graphical abstract

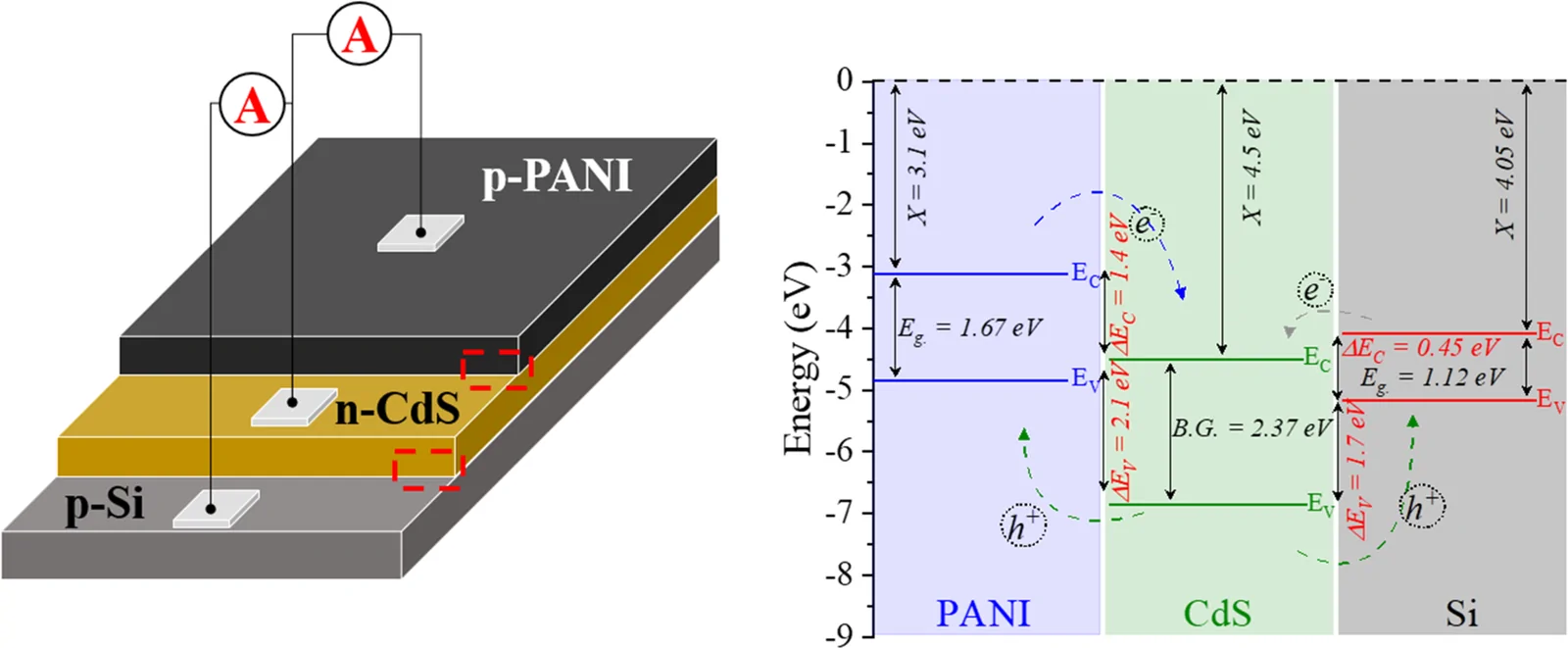

## 1. Introduction

Photodetectors are substantially important components of modern optoelectronics owing to their wide-reaching applications in intelligent sensing, imaging, optical communication, *etc.*^1–3^ The growing need for highly functional photodetectors has encouraged extensive research on the design of photodetectors with special features, such as rapid response speed, excellent operational stability, multi-functional wavelengths, low operating voltage and high figures of merit.^4–6^ Subsequently, semiconductor heterostructured devices have evolved as one of the most effective frameworks for carrier separation enhancement and reduced recombination losses;^7^ for instance, CuFe_2_O_4_–ZnO nanocomposites have been demonstrated for photodetector applications with pronounced figures-of-merit performance in the visible region.^8^ Herein, silicon (Si)-based optoelectronic devices remain the dominant strategy due to their well-established fabrication processes, strong operation in the NIR region, long carrier diffusion length, and excellent carrier mobility.^9,10^ However, the performance remains limited to the NIR region and is weakened throughout the Vis and UV spectral regions. Integrating Si with a semiconductor could realize a high-performance broadband optoelectronic design with a considerable separation rate of photogenerated electron–hole pairs.^11^ Among a number of II–VI semiconductors, cadmium sulfide (CdS) is widely considered because of its direct bandgap (∼2.3 eV), low fabrication cost, chemical stability, and relatively high absorption coefficient;^12,13^ such features make CdS well-suited for UV and Vis wavelength detection. Despite the satisfactory optoelectronic features of CdS, its inherent toxicity, along with environmental concerns, remains a major constraint among the research community, particularly due to potential Cd release.^14^ Incorporating CdS on p-Si results in the formation of a built-in electric field that efficiently separates photogenerated electron–hole pairs and, in turn, leads to an extended spectral response of the intended photodetector design. Moreover, conductive polymers, such as polyaniline (PANI), have also emerged as capable photo-active materials because of their adjustable optical bandgap, tunable conductivity, easy solution processing, mechanical flexibility, *etc.* Among these polymers, PANI is widely considered due to its efficient hole transport and relatively strong Vis spectrum absorption.^15,16^ Integrating PANI into an inorganic semiconductor heterojunction not only extends the incident photon's absorption but also facilitates suppressed carrier recombination along strong charge separation. Despite these promising features, the comparatively low mobility of PANI requires its incorporation along with a highly conductive inorganic semiconductor to achieve its full optoelectronic potential.^17,18^

While significant progress has been made on exploring p-PANI/n-Si, n-CdS/p-Si, and p-PANI/n-CdS heterostructures independently, reports on the p-PANI/n-CdS/p-Si dual-heterostructure remain limited. Herein, localized laser-assisted PANI deposition enables the formation of a second photoactive interface, allowing the p-PANI/n-CdS and n-CdS/p-Si junctions to function independently. This dual-heterostructure allows wavelength-selective photo-detection ranging from UV-Vis to NIR. This work elucidates a systematic p-PANI/n-CdS/p-Si dual-heterostructured photodetector fabrication procedure using pulsed laser deposition (PLD) for CdS deposition along with localized laser-assisted PANI layer growth. The attained dual-heterojunction photodetector demonstrated binary wavelength detection in both the near-UV and visible regions, along with considerable performance in the NIR region due to p-Si functionality.

## 2. Experimental procedures

In a typical procedure, a p-type Si wafer was consecutively cleaned using deionized water, ethanol, and acetone, then dried under an N_2_ flow. Herein, 5 tons-mechanically pressed CdS (99.99%, Sigma-Aldrich) was used to deposit a CdS crystalline layer on two-thirds of the initial p-type Si wafer using the pulsed laser deposition (PLD) approach at room temperature (Fig. 1). In detail, a Q-switched Nd:YAG laser with a wavelength of 532 nm, energy of 200 mJ, and substrate-to-target distance of 6 cm was used; other deposition parameters were maintained at 500 laser pulses, a 6 Hz repetition rate, and a pulse duration of 9 ns. Subsequently, the attained CdS/Si geometry was subject to an annealing process at 200 °C for 15 minutes. Separately, a 0.2 wt% PANI solution was obtained through the dispersion of 0.2 g of PANI (Sigma-Aldrich) in 20 mL of *N*,*N*-dimethylformamide (DMF, Labscan RCI) under continuous stirring at 60 °C for several hours. One-third of the n-CdS/p-Si geometry was then immersed in the PANI solution along with continuous localized laser (405 nm and 1 watt) irradiation focused on the selected third of the CdS layer, with a laser spot size of 0.5 cm^2^ for 20 minutes; this, in turn, allowed selective PANI deposition. Herein, the localized deposition originates from the laser-induced photochemical as well as photothermal phenomena, which promote polymer deposition selectively on the exposed CdS layer. Finally, the deposited PANI layer was gently rinsed with ethanol to remove any undesired adhered polymer, then dried at 50 °C for 10 minutes. The attained layers demonstrated thicknesses of 862 nm, 789 nm, and 1130 nm for Si, CdS, and PANI, respectively (inset in Fig. 1). The attained thickness of both deposited layers was governed by the adopted deposition parameters rather than being predetermined values. To complete the photodetector heterostructured geometry, Ag metal contacts were deposited on each layer (p-PANI/n-CdS/p-Si) using the thermal evaporation method. Based on the expected work function, the deposited Ag electrodes are considered to form Schottky contacts along the semiconductor layers.

**Fig. 1:**
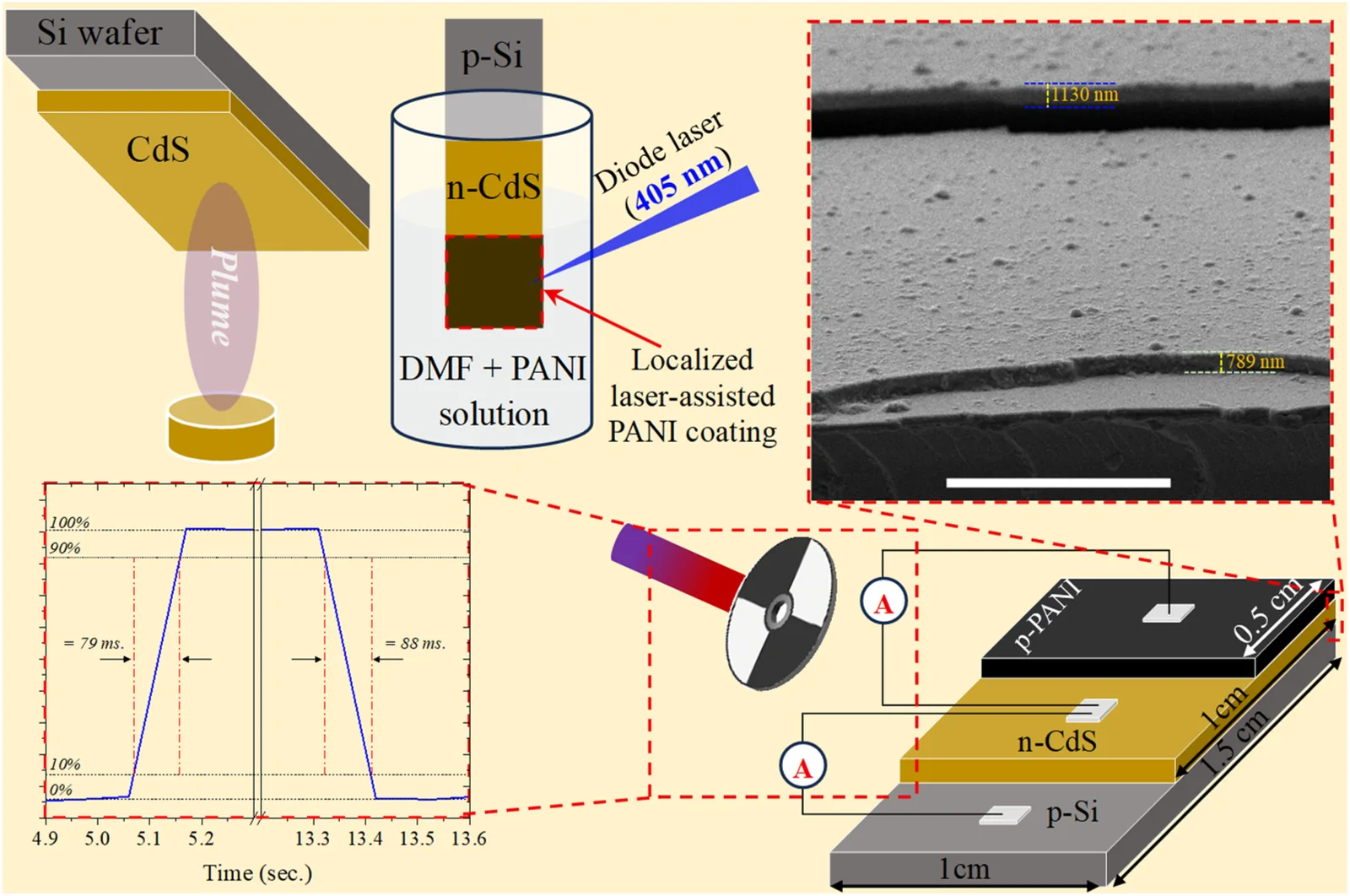
Schematic of the applied methodology and a cross-sectional view with a scale bar of 10 µm.

The microstructural characteristics of the deposited layers were examined using the X-ray diffraction (XRD, PANalytical) technique, while the surface morphology as well as layer thickness were investigated *via* field-emission scanning electron microscopy (FE-SEM, SU8700). The optical investigation was accomplished with a UV-Vis-NIR spectrophotometer (UV-1280, Shimadzu, Japan), while the optical bandgap was attained using the Tauc relation. The Hall-effect measurements were performed with a DX-60, confirming their p- and n-type conductivity based on the Hall coefficient; in this, the deposited layers exhibited mobilities of around 0.11 cm^2^ V^−1^ s^−1^ and 3.9 cm^2^ V^−1^ s^−1^ and hole/electron concentrations of 0.95 × 10^14^ and 1.2 × 10^14^ cm^−3^ for PANI and CdS, respectively. The current–voltage characteristics of the fabricated heterojunction photodetector were evaluated with a source measure unit (Keithley 2401, USA) in conjunction with narrow bandpass filters. Concurrently, the time-resolved characteristics were considered from 10% to 90% with a histogram search approach.

## 3. Results and discussion

The XRD patterns (Fig. 2a) reveal the successful formation of CdS and PANI layers. In detail, the CdS layer (green spectrum) demonstrated diffraction patterns at 2*θ* ≈ 24.799°, 26.541°, 28.340°, and 43.493° corresponding to the (100), (002), (101), and (110) planes, respectively; these, in turn, were found to be in good accordance with hexagonal CdS JCPDS data report no. 41-1049.^19,20^ The PANI layer (blue spectrum) demonstrates binary broad peaks centered around 2*θ* ≈ 15.452° and 25.382°; these are characteristic of semi-crystalline polyaniline. The second peak could correspond to the polymer backbone (π–π stack). Moreover, the deposited CdS shows a strong absorption phenomenon throughout the UV and Vis regions, with a cut-off phenomenon at around 500 nm (Fig. 2b). Conversely, the PANI layer exhibits wide optical absorption ranging from UV to NIR. The inset in Fig. 2b reveals the optical bandgaps of the deposited layers with values of 2.37 eV and 1.67 eV, respectively, estimated using the Tauc relation.^21,22^ The top-view FE-SEM topographies of the deposited layers are presented in Fig. 2c and d. In detail, the CdS layer (Fig. 2c) demonstrates a homogeneous, pinhole-free, and compact surface with nearly perfect coverage, which indicates uniform CdS growth under the PLD technique. Moreover, the PANI layer (Fig. 2d) exhibits a granular and rough morphology and is composed of compactly scattered agglomerates of the utilized polymer; this result suggests an enhanced interfacial rough area. The average nanoparticle diameters were found to be 56.422 ± 7.9 nm and 19.675 ± 4.8 nm based on the statistical analysis of around 100 individual particles, respectively (see insets in Fig. 2c and d).

**Fig. 2:**
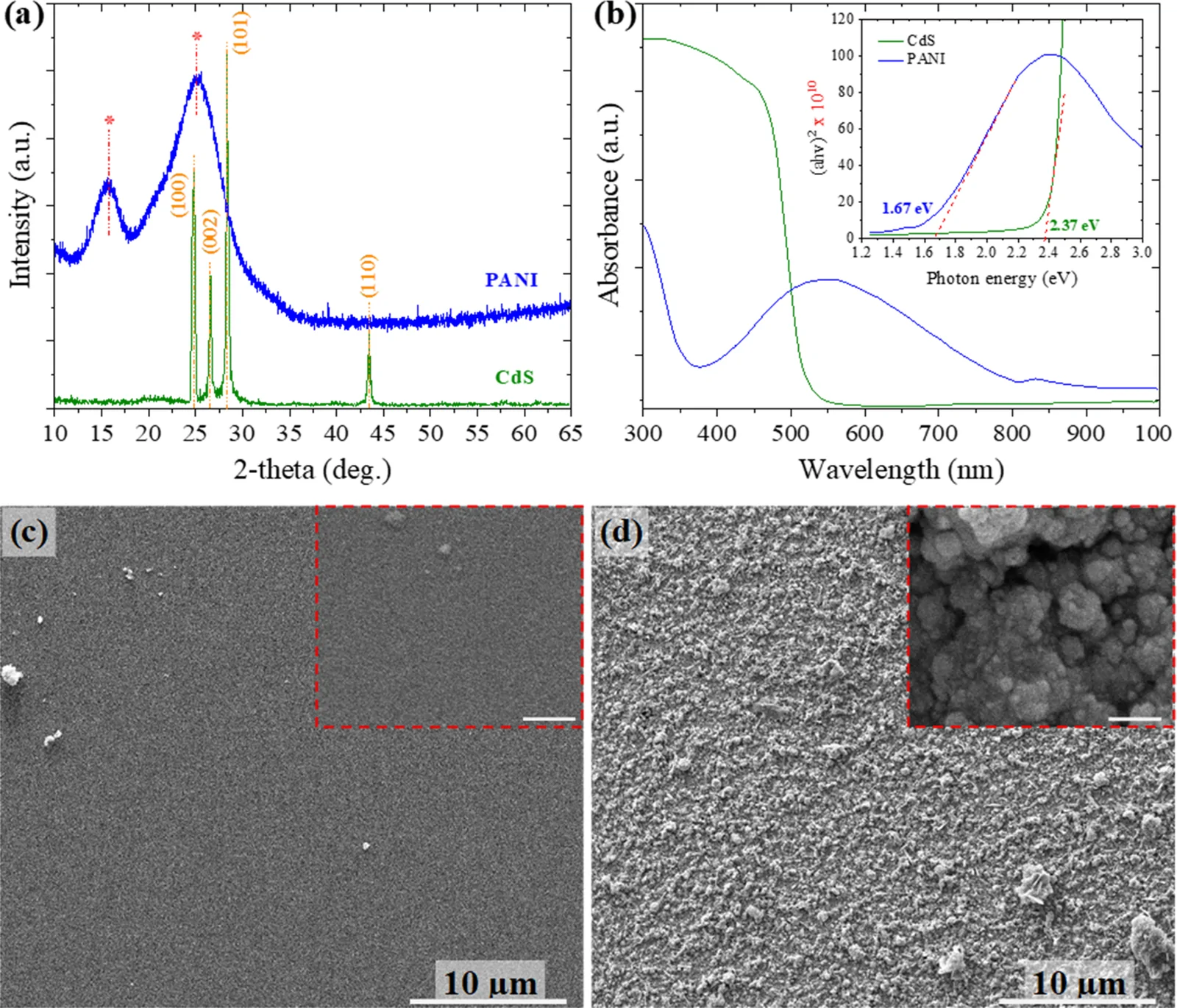
(a) XRD patterns and (b) optical characteristics of the deposited CdS and PANI layers. Top-view FE-SEM topographies of the deposited (c) CdS and (d) PANI, with the insets showing the corresponding higher-magnification FESEM images with a 500 nm scale bar.

The dark *I*–*V* characteristics (insets in Fig. 3a and b) demonstrated a clear diode-like unsymmetrical trend with clear rectification behavior. The dark *I*–*V* characteristics of the proposed dual-junction displayed a pronounced rectification behavior for both considered junctions, with rectification ratios of 6.7 and 9.1 at 5 V for n-CdS/p-Si and p-PANI/n-CdS, respectively. In detail, the p-PANI/n-CdS heterostructure delivered a higher ratio, which indicates a considerably lower reverse leakage current, allowing higher noise suppression.^23^ Despite the relatively low dark current in the p-PANI/n-CdS junction, the forward current of the n-CdS/p-Si heterostructure was found to be much higher. This can be clearly perceived in the resultant *I*_photo_/*I*_dark_ profile (Fig. 3c). The attained reverse leakage current could result from surface states and interfacial defects, which, in turn, allow trap-assisted transport, with respect to the bulk conduction.^24,25^ Upon illumination (Fig. 3a and b), the demonstrated junctions indicated a clear wavelength-dependent photo-response in both reverse and forward directions, with respect to the wavelength utilized. The p-PANI/n-CdS heterostructure demonstrated a maximum photo-response of 5.5 µA at around 460 nm. Such an observation suggests that the addressed interface is highly active within the UV/blue visible region, wherein both utilized layers play crucial roles in incident photon absorption. Conversely, the n-CdS/p-Si heterojunction produced a considerably higher photo-response along the scanned wavelengths, with a maximum value at 530 nm; this noticeable behavior is mainly due to effective carrier separation at the depletion region as well as the photocarrier generation efficiency in Si and CdS. Similar observations were noted for the photo-responsivity [*R*_*λ*_ = (*I*_Photo_)/*P*_in_] (Fig. 3d) and photo-detectivity [D* = *R*_*λ*_(*A*)^1/2^/(2*qI*_dark_)^1/2^] (Fig. 3e) profiles;^26–28^ the investigated active area of the photodetector was 1 cm^2^, while the attained dark current was found to be 8.18 and 0.91 µA for p-PANI/n-CdS and n-CdS/p-Si, respectively. In particular, the p-PANI/n-CdS junction exhibited *R*_*λ*_ and D* of 45.82 mA W^−1^ and 8.5 × 10^10^ Jones at 460 nm, respectively, indicating that this junction is most sensitive to the addressed wavelength because of the combined CdS and PANI optical behavior. Beyond this wavelength, the photo-response gradually decreased, which could be attributed to weaker absorption of CdS after its band edge, while PANI contributes less to the separation of carriers. Meanwhile, the n-CdS/p-Si junction demonstrated values of 417.75 mA W^−1^ and 2.58 × 10^11^ Jones at 530 nm, respectively, along with substantially superior photo-response over the scanned spectral wavelength. Additionally, the n-CdS/p-Si device demonstrated another strong response at 808 nm, confirming its capability for extended NIR photo-detection due to the window effect of the Si wafer.^29^ The external quantum efficiency [EQE = 1240 *R*_*λ*_/*λ* (nm)]^27^ exhibited similar behavior to that of EQE, with values of 12.35% and 97.74% for p-PANI/n-CdS at 460 nm and n-CdS/p-Si at 530 nm (Fig. 3f), respectively. The p-PANI/n-CdS device exhibited a lower EQE trend because of the lower carrier mobility of PANI, greater recombination levels, and higher series resistance at the semiconductor/polymer interface.^30,31^ Overall, the proposed p-PANI/n-CdS/p-Si dual-junction architecture exhibited multi-wavelength detection with a wavelength-selective interface provided by p-PANI/n-CdS and high *R*_*λ*_ performance provided through the n-CdS/p-Si junction.

**Fig. 3:**
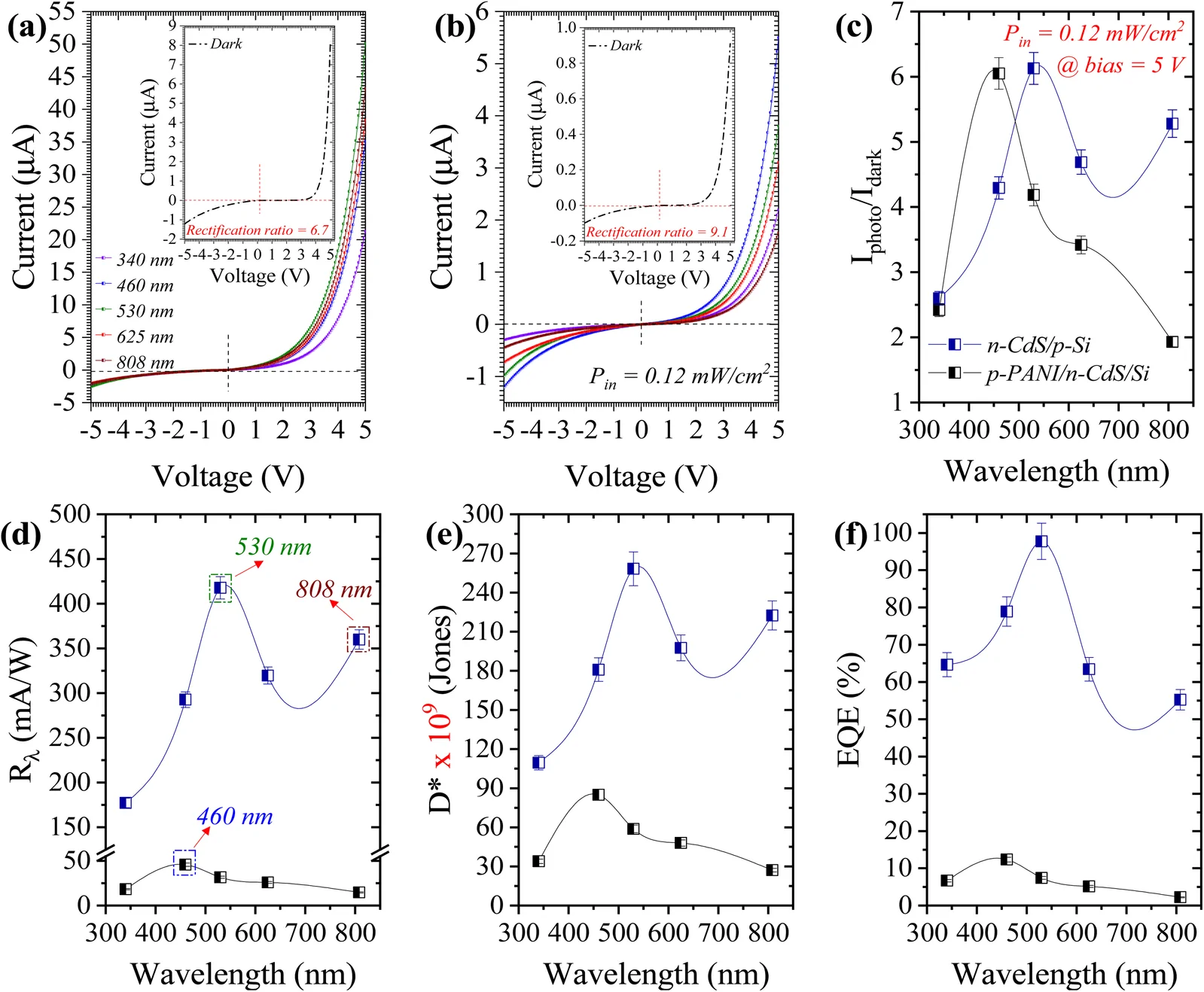
Wavelength-dependency profiles: (a) *I*–*V* curves of n-CdS/p-Si, (b) *I*–*V* curves of p-PANI/n-CdS, and (c) *I*_photo_/*I*_dark_, (d) photo-responsivity, (e) photo-detectivity, and (f) external quantum efficiency curves.

The incident power dependency (Fig. 4) further confirms the considerable photo-response along with wide dynamic sensitivity of the proposed dual-junction device. The p-PANI/n-CdS demonstrated a monotonically increased photocurrent at 460 nm, reaching 17.07 µA as the incident power increased to 7 mW cm^−2^ (Fig. 4a); this supports an efficient photogeneration at the PANI/CdS interface. A similar yet boosted behavior was attained at the n-CdS/p-Si interface (Fig. 4b) with an increase in the photocurrent from 50.13 µA to 136.30 µA as the incident power was increased from 0.12 mW cm^−2^ to 7 mW cm^−2^. This observation indicates that the higher ratio of carrier generation and extraction at the CdS/Si heterointerface is influenced by the superior charge-transport character of the Si wafer and considerable absorption of the CdS layer near the incident 530 nm wavelength. Such singularity was further confirmed by the *I*_photo_/*I*_dark_ profile (Fig. 4c). The investigated *R*_*λ*_ and D* (Fig. 4d and e) demonstrated descending trends for both junctions, with values reduced to 2.44 mA W^−1^ and 4.5 × 10^9^ Jones for p-PANI/n-CdS and 192.47 mA W^−1^ and 1.2 × 10^10^ Jones for n-CdS/p-Si, respectively. The inverse figures-of-merit dependency as a function of incident power is well-established for photodetectors governed by the inversely proportional relationship between *R*_*λ*_ and incident power (*R*_*λ*_ ∝ *P*_in_^−1^).^32^ Additionally, progressive filling of localized states as well as deep trap-assisted recombination could also play vital roles in such phenomenon.^33^ At low incident power, high gain is acquired as a result of the photogenerated carriers' separation by the built-in electric field. However, as the illumination increased, deep traps become saturated and recombination likelihood grows; this allows a decrease in the photocurrent per unit optical power. The EQE (Fig. 4f) revealed values as low as 2.25% and 55.24% at 7 mW cm^−2^ for p-PANI/n-CdS and n-CdS/p-Si, respectively. The closely linear fitting demonstrated in the power-dependent schemes, along *R*^2^ ≈ 1, indicates stable and reproducible photoconversion over the examined power range, while the sublinear increase in photocurrent reflects the occurrence of trap-mediated carrier transport and recombination centers.

**Fig. 4:**
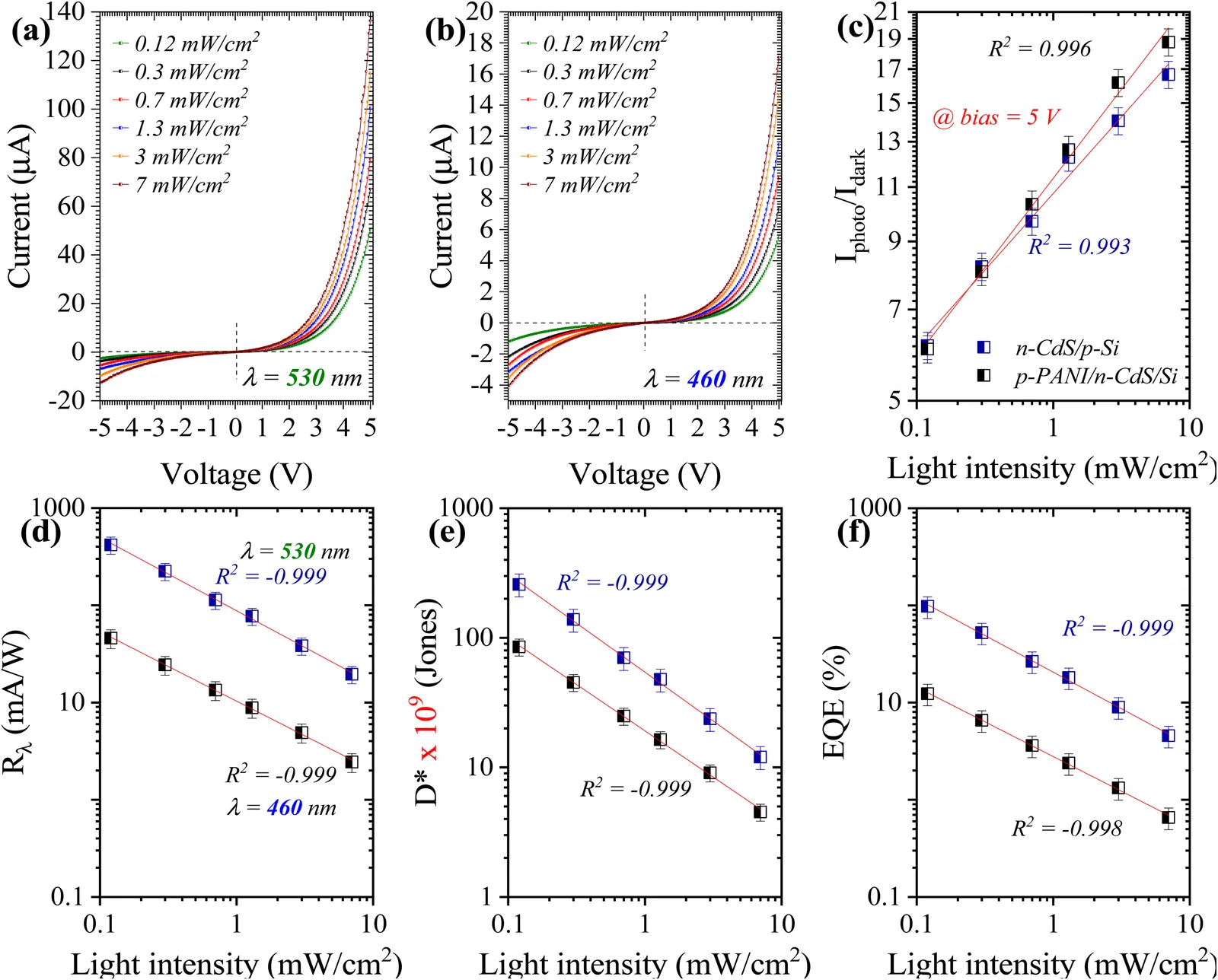
Incident light intensity profiles: (a) *I*–*V* curves of n-CdS/p-Si, (b) *I*–*V* curves of p-PANI/n-CdS, and (c) *I*_photo_/*I*_dark_, (d) photo-responsivity, (e) photo-detectivity, and (f) external quantum efficiency curves.

The time-resolved behavior of the proposed dual-heterostructure device exhibits excellent switching reproducibility and operational stability over periodic illumination cycles (15 cycles). Both investigated heterojunctions at 460 nm and 530 nm exhibited highly repeatable transitions between the on/off states, without traceable signal degradation (Fig. 5a and c); the current demonstrated an immediate increase reaching its maximum value, after which it returned to its initial level in the dark. This almost identical on/off behavior indicates the excellent reversibility of the fabricated dual-heterostructured device. In terms of response/recovery speed (see inset in Fig. 5a and c), the fabricated junctions exhibited rather rapid switching, with values of 82/87 ms and 79/88 ms, respectively. The faster rise time as compared to the recovery suggests faster electron injection than recombination.^34^ In addition, the p-PANI/n-CdS junction delivered a faster response than the n-CdS/p-Si junction. Such singularity suggests that localized laser-assisted polymer deposition forms a sufficiently intimate polymer/semiconductor interface that facilitates well-organized charge transfer while preserving fast carrier extraction. The response speed could be limited by carrier trapping at the interface/defect states as well as by carrier transit through the PANI film, where photocurrent recovery could also be affected.^35^ The long-term stability test for both junctions confirms an excellent operational robustness over 4 weeks. In detail, the n-CdS/p-Si photodetector retained over 87% of its initial performance (Fig. 5b), while p-PANI/n-CdS demonstrated 93.7% performance durability (Fig. 5d). The slight performance reduction over 4 weeks could be due to a gradual trapping of carriers and a slight interfacial relaxation over frequent incident power application rather than active material degradation.^36^Table 1 provides a comparison of the data against reported data.

**Fig. 5:**
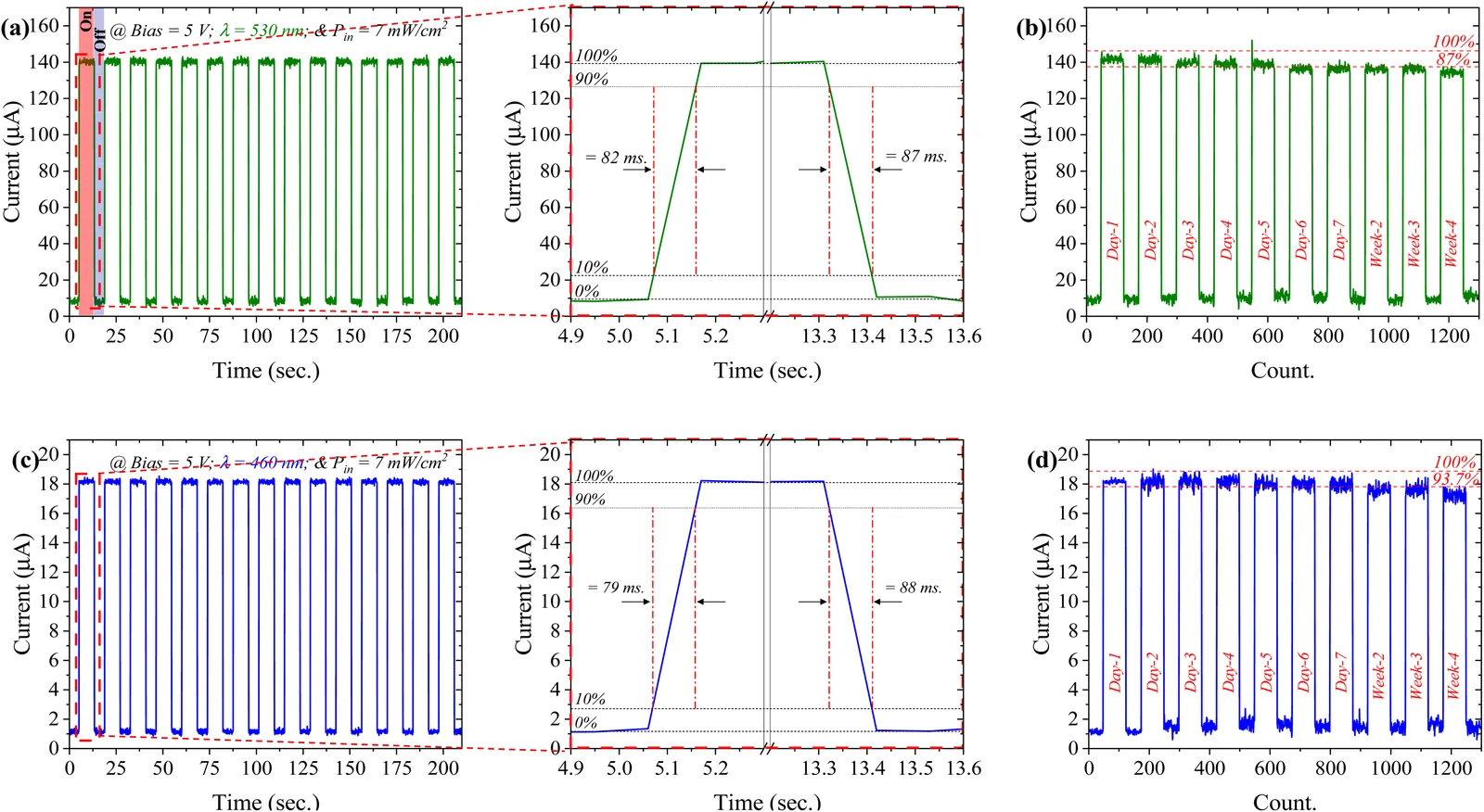
Time-resolved characteristics of the fabricated p-PANI/n-CdS/p-Si dual-heterostructured photodetector: (a) and (c) on/off behavior and (b) and (d) long-term stability tests. Green represents the n-CdS/p-Si, while blue refers to p-PANI/n-CdS.

**Table 1:** Performance comparison of the fabricated p-PANI/n-CdS/p-Si dual-heterojunction along recently reported similar geometries

Structure	Wavelength (nm)	Method	*R*_*λ*_ (mA W^−1^)	D* (Jones)	*T*_rise_/*T*_fall_ (ms)	Ref.
PANI/CdS	360	Spray	4.86	4.0 × 10^10^	0.06/0.06	37
ZnO/PANI	400	Coating/RF	39	—	—	38
CdS/Si	525	PLD	48	—	112–119	20
CdS/Si	—	Magnetron sputtering	460	4.09 × 10^11^	—	39
CdS/Si	530	PLD	417.75	2.58 × 10^11^	82/87	This work
PANI/CdS	460	Localized laser	45.82	8.5 × 10^10^	79/88	This work

The mechanism of carrier transport for the proposed p-PANI/n-CdS/p-Si dual-heterojunction was further interpreted using the Anderson model.^40^ Specifically, the adopted electron affinities of PANI, CdS, and Si were 3.1, 4.5, and 4.05 eV, respectively, corresponding to VB maxima located at −4.77, −6.87, and −5.17 eV. Moreover, the p-PANI/n-CdS interface exhibits Δ*E*_C_ = 1.4 eV and Δ*E*_V_ = 2.1 eV, while the n-CdS/p-Si interface demonstrates Δ*E*_C_ = 0.45 eV and Δ*E*_V_ = 1.7 eV. Herein, the established offsets allow energetically favorable photogenerated carrier pathways where electrons are transferred toward n-CdS from both p-type materials, while holes are driven toward p-PANI and p-Si. This carrier separation, across the fabricated interfaces, suppresses interfacial recombination for the p-PANI/n-CdS/p-Si dual-heterojunction photodetector (Fig. 6).

**Fig. 6:**
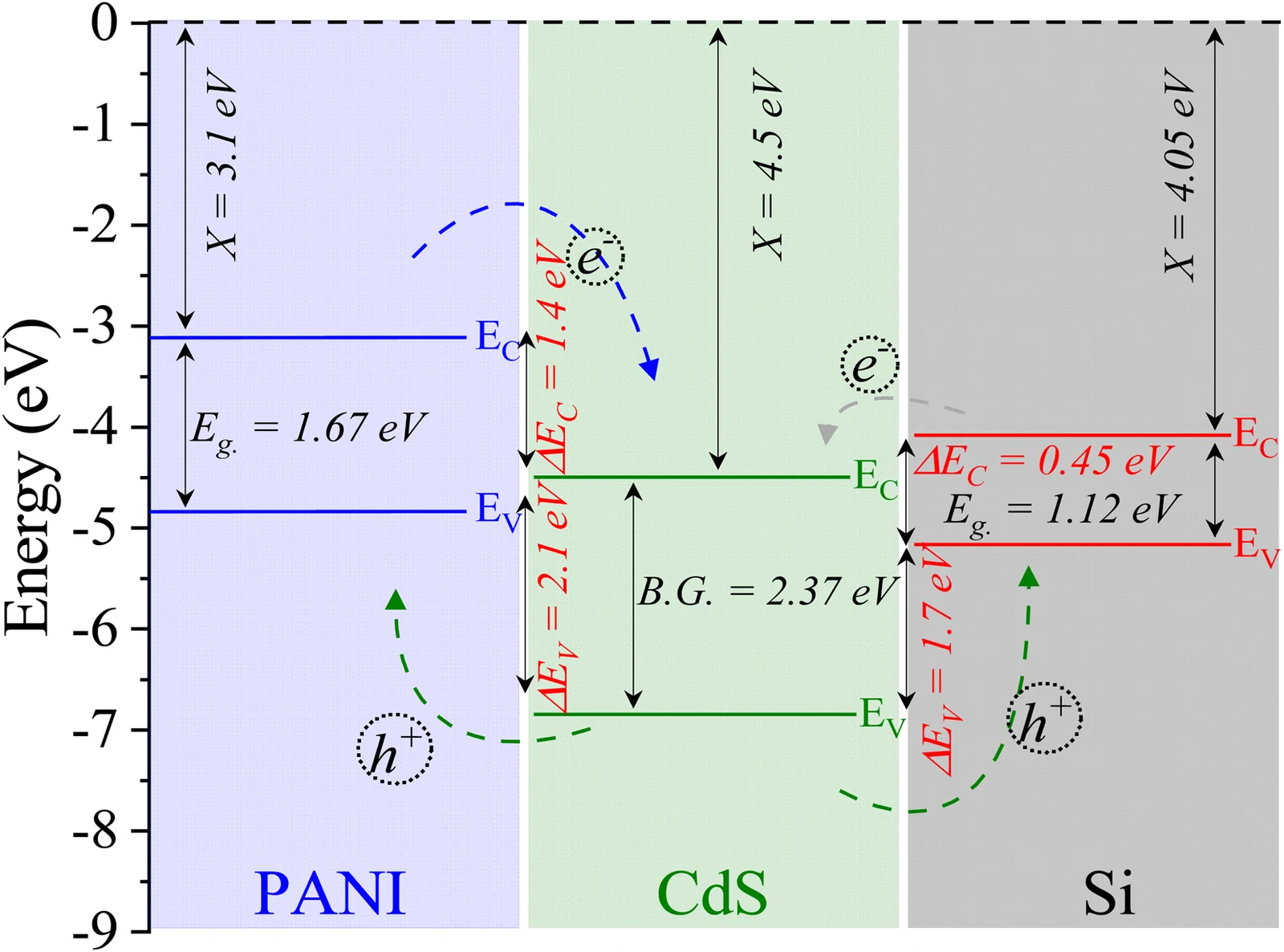
Proposed bandgap alignment for the fabricated p-PANI/n-CdS/p-Si dual-heterojunction.

## 4. Conclusion

A novel p-PANI/n-CdS/p-Si dual-heterostructured photodetector was systematically fabricated *via* PLD of CdS and localized laser-assisted PANI on a Si wafer. This allowed the formation of independent binary active interfaces within a single geometry; the resultant device exhibited pronounced performance in the UV, Vis, and NIR regions. The p-PANI/n-CdS heterostructure delivered its highest performance at 460 nm, while n-CdS/p-Si exhibited remarkable dual functionality at 530 nm and 808 nm. The fabricated heterojunctions demonstrated considerable rectifying profiles, with values reaching 9.1 and 6.7, while maximum *R*_*λ*_ values of 45.82, 417.75, and 359.96 mA W^−1^ were attained at 460 nm, 530 nm, and 808 nm, respectively, with respect to the junction considered. In terms of incident power, both junctions presented excellent linearity (*R*^2^ ≈ 1). The dual-heterostructure confirmed quick response/recovery trends, with values of 79/88 ms and 82/87 ms, while maintaining 93.7% and 87% of their initial performance for p-PANI/n-CdS and n-CdS/p-Si, respectively, over four weeks.

## Conflicts of interest

There are no conflicts to declare.

## Data Availability

All data used are presented in the manuscript.
